# Rabbit hemorrhagic disease virus capsid, a versatile platform for foreign B-cell epitope display inducing protective humoral immune responses

**DOI:** 10.1038/srep31844

**Published:** 2016-08-23

**Authors:** Noelia Moreno, Ignacio Mena, Iván Angulo, Yolanda Gómez, Elisa Crisci, María Montoya, José R. Castón, Esther Blanco, Juan Bárcena

**Affiliations:** 1 Centro de Investigación en Sanidad Animal (INIA-CISA), Valdeolmos, 28130 Madrid, Spain; 2 Centre de Recerca en Sanitat Animal (CReSA), UAB-IRTA, Campus de la Universitat Autònoma de Barcelona, Bellaterra, 08193 Barcelona, Spain; 3 The Pirbright Institute, Ash Road, Woking, Surrey, GU24 0NF, United Kingdom; 4 Department of Structure of Macromolecules, Centro Nacional de Biotecnología/CSIC, Cantoblanco, 28049 Madrid, Spain

## Abstract

Virus-like particles (VLPs), comprised of viral structural proteins devoid of genetic material, are tunable nanoparticles that can be chemically or genetically engineered, to be used as platforms for multimeric display of foreign antigens. Here, we report the engineering of chimeric VLPs, derived from rabbit hemorrhagic disease virus (RHDV) for presentation of foreign B-cell antigens to the immune system. The RHDV capsid comprises 180 copies of a single capsid subunit (VP60). To evaluate the ability of chimeric RHDV VLPs to elicit protective humoral responses against foreign antigens, we tested two B-cell epitopes: a novel neutralizing B-cell epitope, derived from feline calicivirus capsid protein, and a well characterized B-cell epitope from the extracellular domain of influenza A virus M2 protein (M2e). We generated sets of chimeric RHDV VLPs by insertion of the foreign B-cell epitopes at three different locations within VP60 protein (which involved different levels of surface accessibility) and in different copy numbers per site. The immunogenic potential of the chimeric VLPs was analyzed in the mouse model. The results presented here indicated that chimeric RHDV VLPs elicit potent protective humoral responses against displayed foreign B-cell epitopes, demonstrated by both, *in vitro* neutralization and *in vivo* protection against a lethal challenge.

Nanobiotechnology involves the exploitation of biomaterials, devices or methodologies at the nanoscale. Virus particles constitute natural nanomaterials that are receiving increasing attention due to their potential use in diverse biomedical applications, such us cell targeting, drug delivery or vaccine development^1^,^2^,^3^,^4^,^5^.

Virus-like particles (VLPs) are supramolecular assemblages with well-defined geometry that mimic the overall structure of native virions, while lacking any viral genome^6^,^7^. These multimeric protein cages are based on the natural intrinsic ability of structural viral subunits to spontaneously self assemble into nanoparticles (in the range of 25–100 nm), when produced using recombinant expression systems^8^,^9^. They are composed of multiple copies of one or more viral proteins and are usually antigenically indistinguishable from infectious virus or subviral particles^10^. Thus, VLPs exhibit properties that are highly advantageous for vaccine development, combining convenient features of whole-virus-based and recombinant subunit vaccines^11^. By their nature, VLP-based vaccines provide a high safety profile, can stimulate both innate and adaptive immune responses, elicit protective systemic and mucosal immunity, and have been shown to exhibit self-adjuvanting abilities^12^,^13^,^14^. These characteristics have made VLPs attractive stand-alone vaccine candidates for many viral diseases^15^. In addition, VLPs can be chemically or genetically engineered^16^,^17^,^18^,^19^,^20^ to be used as platforms for multimeric display of foreign antigens derived from viruses or other pathogens, which, in turn, can be developed into vaccines^21^,^22^. Here, we report the generation of chimeric VLPs displaying foreign B-cell antigens to the immune system, using as platform the capsid of rabbit hemorrhagic disease virus (RHDV).

Rabbit haemorrhagic disease (RHD) is a highly infectious and fatal disease of the European rabbit (*Oryctolagus cuniculus*) (reviewed in^23^). The etiological agent, RHDV, is the prototype species of *Lagovirus* genus within the *Caliciviridae* family. It is a non-enveloped, icosahedral, single-stranded positive-sense RNA virus. The virus capsid (~40 nm diameter) comprises 180 copies (90 dimers) of a single capsid subunit, the VP60 protein (also termed VP1), arranged with T = 3 symmetry to form 12 pentamers and 20 hexamers. RHDV, as most caliciviruses, cannot be grown in cell culture and much of our understanding of these viruses relies on studies performed with recombinant VLPs that are morphologically and antigenically identical to infectious RHDV virions^24^. RHDV VLPs have been shown to induce full protection of rabbits against a lethal challenge with RHDV and are used as diagnostic reagents^24^,^25^.

The VP60 protein has three domains^26^ (Fig. 1a), an N-terminal arm (NTA), a shell (S) forming a scaffold which protects the viral RNA, and a flexible protruding domain (P) at the capsid surface, which contains determinants for virus-host receptor interactions and antigenic diversity^25^,^26^,^27^. The P domain can be further divided into P1 and P2 subdomains, with P2 subdomain located at the outermost surface-exposed region of the viral capsid.

We have previously performed an exhaustive structural analysis of the RHDV capsid and have shown that the VP60 protein can accommodate insertions of foreign amino acid sequences without disrupting VLP formation^28^,^29^, raising the possibility of using RHDV VLPs as foreign epitope carriers for vaccine development. In this report we evaluated the ability of chimeric RHDV VLPs to elicit humoral responses against inserted foreign B-cell epitopes. We tested two different epitopes: a newly described neutralizing B-cell epitope derived from the feline calicivirus (FCV) capsid protein (VP62), and the well characterized and highly conserved B-cell epitope located within the extracellular domain of the influenza A virus M2 protein (M2e)^30^. We generated sets of chimeric RHDV VLPs by inserting the foreign B-cell epitopes at different locations within the VP60 protein and in different copy numbers per site. The immunogenic potential of the different chimeric VLPs was analyzed in the mouse model. The results indicated that chimeric RHDV VLPs elicit potent protective humoral responses against foreign B-cell epitopes, demonstrated by both, *in vitro* neutralization and *in vivo* protection against a lethal challenge.

## Results

### Generation and characterization of RHDV chimeric VLPs displaying an FCV capsid protein-neutralizing epitope

**Figure 1:**
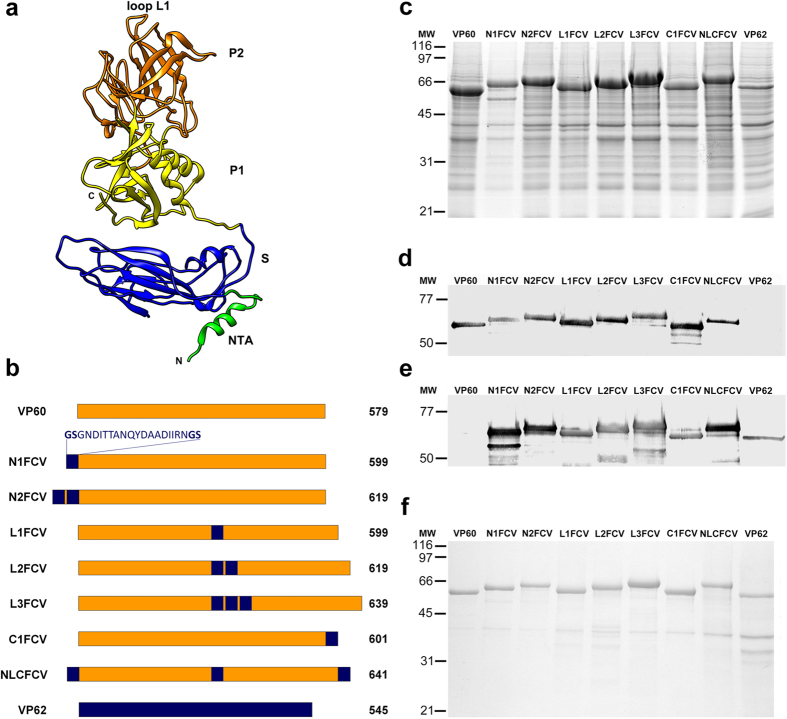
Expression and characterization of VP60 insertion mutants harbouring a FCV capsid protein B-cell epitope. **(a)** Ribbon representation of the VP60 protein structure (Protein Data Bank [PDB] accession number 3J1P). The NTA, S domain, P1 and P2 subdomains, and loop L1 are indicated. **(b)** Schematic representation showing names (left) and protein lengths in amino acids (right). The amino acid sequence depicted (FCV B-cell epitope) was inserted at the indicated positions in each VP60 insertion mutant. RHDV capsid protein (VP60) and FCV capsid protein (VP62) are also shown. **(c)** H5 cells were infected with each recombinant baculovirus and infected-cell lysates were analyzed by SDS-10% PAGE. **(d,e)** Western blots performed using a rabbit hyperimmune serum against RHDV to detect VP60 protein **(d)**, or a monoclonal antibody directed against the FCV B-cell epitope **(e)**. **(f)** Infected H5 cell cultures were subjected to VLP-purification procedures and the resulting samples were characterized by SDS-10% PAGE. Molecular weight markers (MW; ×10^3^ Da) are given on the left.

Recombinant baculoviruses expressing different VP60 insertion mutants were generated (Fig. 1b). The foreign amino acid sequence inserted (22 aa) contained a B-cell epitope derived from the FCV capsid protein (VP62). This newly described B-cell epitope was recognized by neutralizing monoclonal antibodies obtained and characterized at our laboratory. The sequence inserted was flanked by amino acids glycine and serine (GS) as a flexible linker intended to facilitate capsid assembly.

The foreign peptide sequence was inserted at three locations within the VP60 protein and in different copy numbers per site (Fig. 1b), on the basis of previous structural analyses^28^,^29^. Chimeric mutants were generated by inserting one or two copies of the B-cell epitope between amino acid positions 2 and 3 of VP60 protein sequence (N1FCV and N2FCV, respectively). According to the atomic structure of RHDV capsid^26^, the N-terminus of VP60 protein is facing the inner core of the viral capsid. Another set of chimeric mutants was produced by inserting 1–3 copies of the B-cell epitope between amino acid positions 306 and 307 of VP60 protein (L1FCV, L2FCV and L3FCV, respectively). This insertion site is located within loop L1 at the tip of the P2 subdomain of VP60 protein, the most surface-exposed region of the viral capsid^26^,^29^. An additional chimeric mutant was generated harbouring one copy of the B-cell epitope at the C-terminal end of VP60 protein (C1FCV), which faces the outer surface of the viral capsid at the pentameric and hexameric cup-shaped depressions^26^,^29^. We completed the set of mutants generating a construct (NLCFCV) bearing one copy of the B-cell epitope in each of the three insertion sites identified within VP60 protein. Finally, along with the RHDV-derived VP60 chimeric mutants, we produced a recombinant baculovirus expressing the native FCV capsid protein (VP62), to enable comparison of the immunogenicity induced by the foreign epitope incorporated to the RHDV chimeric VLPs, with that elicited by the same epitope in its natural context.

Expression of the recombinant constructs in H5 insect cell cultures infected with the corresponding recombinant baculoviruses was analysed by SDS-10% PAGE. As shown in Fig. 1c, extracts from cells infected with the recombinant baculovirus harbouring the RHDV VP60 native protein, exhibited a major protein band with an apparent molecular weight of ≈60 kDa, which was not present in wild-type baculovirus-infected cells (data not shown). Expression levels of the VP60 insertion mutants were grossly similar to those of VP60 native protein, and the corresponding bands were detected with mobilities according to the length of the foreign peptide sequences incorporated in each construct. The FCV VP62 native protein was detected with its expected mobility (≈59.4 kDa), exhibiting lower expression levels than the VP60-derived constructs. A rabbit hyperimmune serum against RHDV specifically detected baculovirus-expressed VP60 protein as well as the VP60 chimeric insertion mutants, but did not react with FCV VP62 protein in Western blot (Fig. 1d). A monoclonal antibody directed against the FCV-derived B-cell epitope reacted both, with native VP62 protein and with the VP60 chimeric constructs, but did not react with native VP60 protein (Fig. 1e).

**Figure 2:**
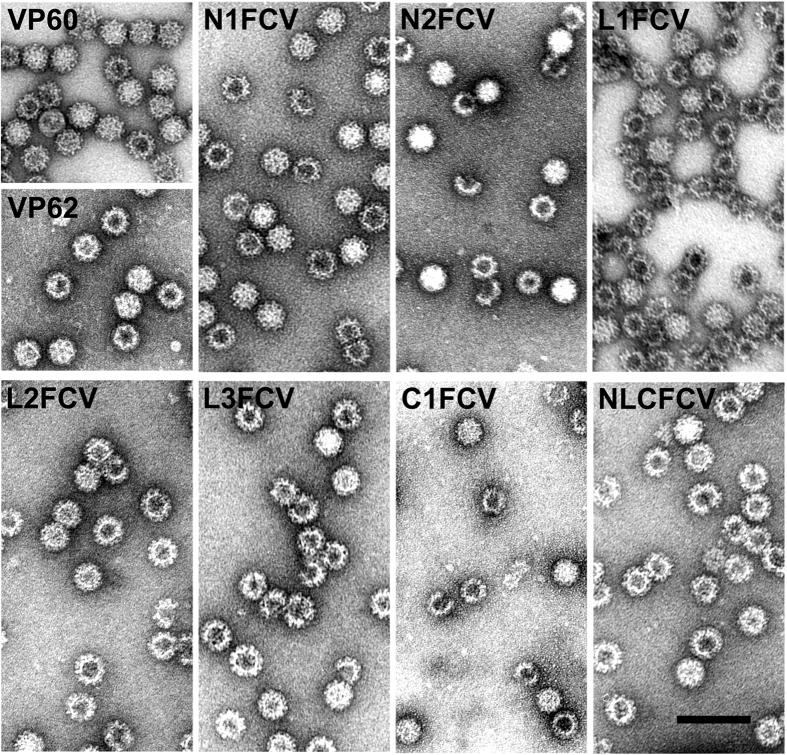
Analysis of VP60-related virus-like particles by electron microscopy. Negatively stained purified particles corresponding to RHDV VP60 and FCV VP62 (top, left), and the indicated VP60 chimeric mutants were analyzed by electron microscopy. Scale bar = 100 nm.

To determine whether the recombinant constructs generated were able to assemble into particulate material, infected H5 cell cultures were subjected to VLP-purification procedures and the resulting samples were characterized by SDS-10% PAGE (Fig. 1f) and electron microscopy (Fig. 2). Negatively stained samples corresponding to all the chimeric constructs generated harbouring the FCV-derived epitope (RHDV-FCV VLPs), assembled as VLPs of approximately 40 nm in diameter, which were morphologically similar to the VLPs formed by wild-type VP60 and VP62 proteins.

### Humoral immune responses to chimeric RHDV-FCV VLPs

**Figure 3:**
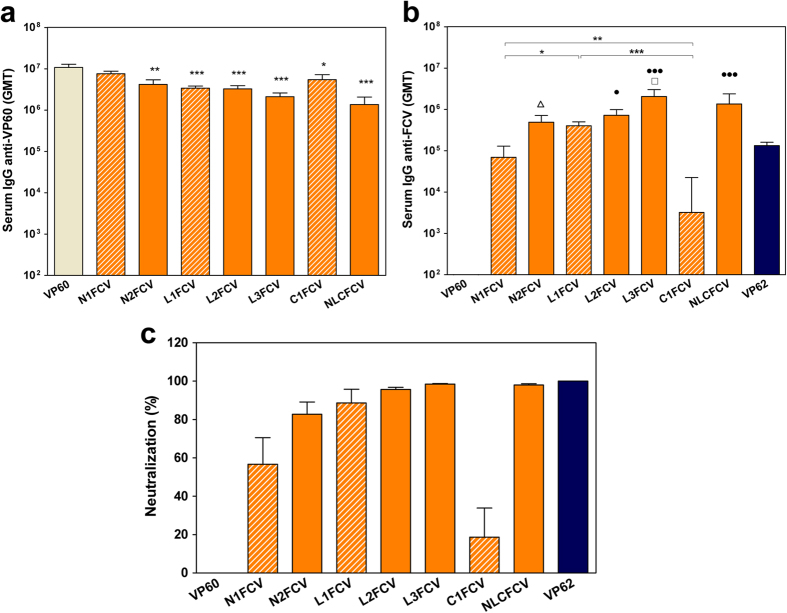
Humoral responses induced in mice by immunization with chimeric RHDV-FCV VLPs. Sera samples from groups of mice inoculated twice with the indicated VLPs were analyzed by ELISA **(a,b)**, or a plaque-reduction neutralization assay specific for FCV virus **(c)**. Serum IgG antibody titers were measured by ELISA using RHDV VLPs to detect anti-VP60 antibodies **(a)**, or a synthetic peptide encompassing FCV B-cell epitope sequence, to detect anti-FCV antibodies **(b)**. The GMT was calculated for each group of mice. Error bars show the standard error of the mean. The groups corresponding to chimeric VLPs harbouring one copy of the inserted epitope per VP60 monomer (N1FCV, L1FCV and C1FCV) are shown as hatched bars. In **(a)**, statistically significant differences in anti-VP60 antibody titers, with respect to those corresponding to the VP60 group are shown as *p < 0.05, **p < 0.01, ***p < 0.001. In **(b)**, statistically significant differences in anti-FCV antibody titters between groups N1FCV, L1FCV and C1FCV (hatched bars) are shown as *; differences between groups harbouring the inserted epitope at the N-terminus are shown as Δ; differences between groups harbouring the inserted epitope at loop L1 are shown as ◻; and differences with respect to the antibody titters corresponding to the FCV VP62 group are shown as ⦁.

Groups of five C57BL/6 mice were immunized twice intraperitoneally with 100 μg of each purified VLP emulsified in Montanide adjuvant. Blood samples were collected 14 days after boost and the sera obtained were tested for antibodies against VP60 protein (RHDV VLPs) (Fig. 3a) and the FCV-derived B-cell epitope (a synthetic peptide) (Fig. 3b). Serum IgG antibody titers were measured by ELISA, and geometric mean titers (GMTs) were calculated for each group of mice. All preimmune serum samples were negative (titer < 50) for IgGs against both antigens tested (data not shown). Control mice only receiving PBS with adjuvant also lacked specific serum IgG (titer < 50 data not shown). As expected, all mice immunized with RHDV or chimeric RHDV VLPs developed high titers of VP60-specific antibodies (GMTs ranging from 1.37 × 10^6^ to 10.75 × 10^6^; Fig. 3a). However, anti-VP60 responses induced by chimeric VLPs with insertions at loop L1 (L1FCV, L2FCV, L3FCV and NLCFCV) were significantly lower (*P* < 0.001) than those induced by native RHDV VLPs (VP60). As shown in Fig. 3b, all mice immunized with either chimeric RHDV VLPs or FCV VLPs (VP62) developed anti-FCV specific antibodies, whereas sera from mice immunized with native RHDV VLPs were negative (titer < 50). Insertion site of the foreign B-cell epitope was shown to influence the anti-FCV antibody titers induced by the chimeric VLPs (Fig. 3b, compare hatched bars). Mice inoculated with L1FCV exhibited significantly higher titers (GMT = 4.04 × 10^5^), as well as lower dispersion in antibody titers within the group (lower standard error of the mean), than mice inoculated with N1FCV (GMT = 6.94 × 10^4^; *P* < 0.05) or mice inoculated with C1FCV (GMT = 3.22 × 10^3^; *P* < 0.001). Interestingly, the GMT of group L1FCV was three-fold higher than that corresponding to the group of mice inoculated with native VP62 FCV VLPs (GMT = 1.32 × 10^5^), although this difference was not statistically significant. The results also revealed that increasing the FCV epitope copy number per VLP resulted in a significant increase in the anti-FCV specific antibody titers elicited (Fig. 3b, compare GMTs of N1FCV with N2FCV, and GMTs of L1FCV with L2FCV, L3FCV and NLCFCV). It is interesting to note that construct N2FCV, incorporating two copies of the foreign epitope per VP60 monomer (360 copies per VLP), induced specific antibody titers (GMT = 4,91 × 10^5^) which were seven-fold higher than those corresponding to N1FCV, displaying half the number of copies of the foreign epitope (180 copies per VLP). Likewise, construct L3FCV (540 copies per VLP) induced specific antibody titers (GMT = 2.06 × 10^6^) five-fold higher than those elicited by L1FCV (180 copies per VLP). This suggested that the increased specific immunogenicity observed with both chimeric constructs harbouring tandem repeats of the foreign epitope, was not merely attributable to a concomitant increase in the amount of epitopes delivered, but was rather a result of a synergistic effect of tandem repeats over the immune response elicited. However, this was not always the case. Constructs L2FCV (GMT = 7.25 × 10^5^) and NLCFCV (GMT = 1.36 × 10^6^) induced specific antibody titers that were approximately two-fold and three-fold those induced by L1FCV, respectively. Thus, a synergistic effect was not evident in those instances. Overall, the highest titers of antibodies against the FCV foreign epitope were induced by chimeric construct L3FCV, which elicited a GMT that was over fifteen-fold higher than that induced by native VP62 FCV VLPs.

### Neutralizing antibody responses induced by chimeric RHDV-FCV VLPs

Sera samples were analyzed by a plaque-reduction neutralization assay (Fig. 3c). Negative control sera (data not shown), as well as sera from the group of mice inoculated with native VP60 RHDV VLPs, failed to induce any detectable neutralizing activity. All mice immunized with chimeric RHDV VLPs exhibited significant levels of anti-FCV neutralizing activity, albeit to different extents (ranging from 18.64% to 98.45% of plaque reduction). These results demonstrated the ability of the FCV B-cell epitope reported in this work to elicit efficient neutralizing antibodies against FCV infection, although a complete neutralizing activity (100% plaque-reduction) was not achieved under the assay conditions used (sera samples diluted 1/20). In contrast, sera samples from mice immunized with native VP62 FCV VLPs exhibited 100% neutralizing activity (even when assayed at 1/80 dilution). This probably reflected the ability of FCV VLPs to induce a polyclonal neutralizing antibody response, with the concurrent involvement of several neutralizing epitopes present at the FCV capsid^31^, while chimeric RHDV VLPs were only displaying a single FCV B-cell epitope. Results presented here on the magnitude of the anti-FCV neutralizing response, regarding foreign-epitope insertion site and epitope copy numbers per VLP monomer, closely resembled those indicated above (compare Fig. 3b,c). Thus, the chimeric construct L1FCV elicited higher neutralizing activity than N1FCV and C1FCV, and increasing epitope copy numbers resulted in an enhancement of specific anti-FCV neutralizing activity.

### Generation and characterization of RHDV chimeric VLPs displaying influenza A virus M2e

**Figure 4:**
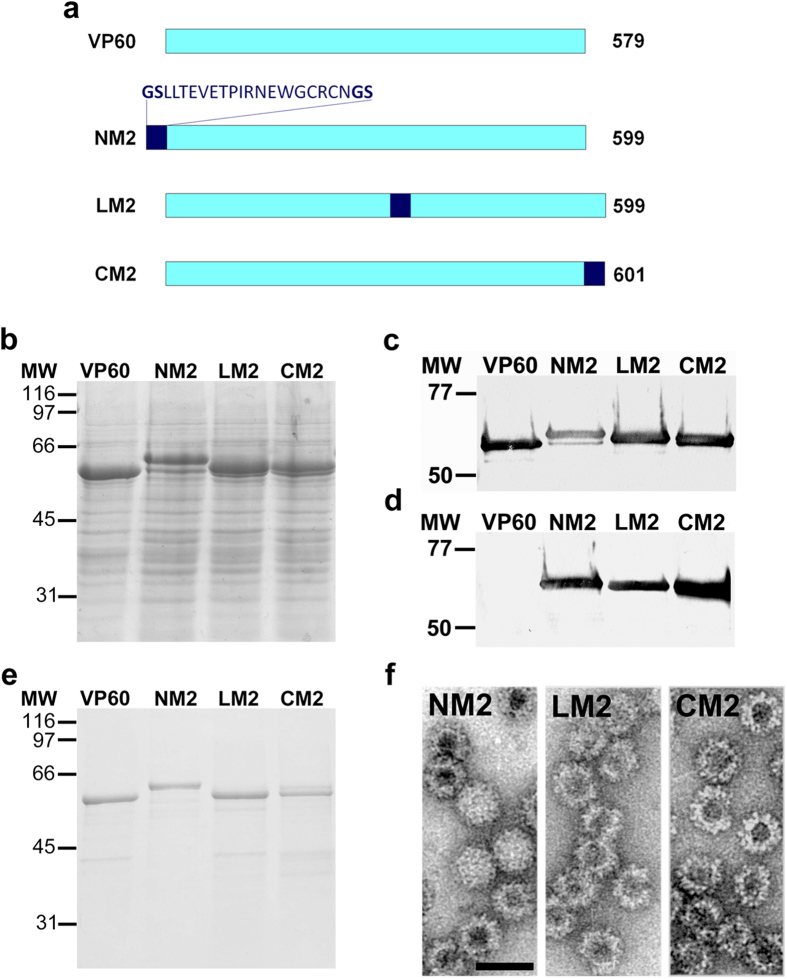
Expression and characterization of VP60 insertion mutants harbouring influenza A virus M2e. (**a**) The scheme shows names (left) and protein lengths in amino acids (right). The amino acid sequence depicted (M2e epitope) was inserted at the indicated positions in each VP60 insertion mutant. **(b)** SDS-10% PAGE analysis of H5 cell-lysates infected with recombinant baculoviruses expressing the indicated VP60 insertion mutants. **(c,d)** Western blots, performed using a rabbit hyperimmune serum against RHDV to detect VP60 protein **(c)**, or a monoclonal antibody directed against M2e **(d)**. **(e)** SDS-10% PAGE analysis of purified VLP preparations. Molecular weight markers (MW; ×10^3^ Da) are given on the left. **(f)** Negatively stained purified particles corresponding to the indicated VP60 chimeric mutants analyzed by electron microscopy. Scale bar = 100 nm.

The promising results obtained led us to further explore the feasibility of using RHDV VLPs as a vaccine delivery system, by analyzing their ability to induce protective immunity against a viral challenge in an animal model. Therefore, we decided to use a well characterized B-cell epitope, the ectodomain of influenza A virus M2 protein (M2e), which is able to render protection against a lethal challenge in mice, if correctly delivered to the immune system^30^. Following the same approach described above, we generated a new set of recombinant baculoviruses expressing VP60 insertion mutants (Fig. 4a). The foreign amino acid sequence was incorporated at the three previously described insertion sites within the VP60 protein, generating the chimeric VP60-M2 constructs: NM2, LM2 and CM2. Infection of H5 insect cell cultures with the corresponding recombinant baculoviruses, resulted in efficient expression of the VP60-M2 proteins (Fig. 4b). Western blot analysis revealed that chimeric constructs were detected by both, a rabbit hyperimmune serum against RHDV (Fig. 4c) and a monoclonal antibody specific for M2e (Fig. 4d). Finally, infected H5 cell cultures were subjected to VLP-purification procedures and the resulting samples (Fig. 4e) were characterized by negative-staining electron microscopy, confirming that the three chimeric constructs assembled into VLPs of approximately 40 nm in diameter (RHDV-M2 VLPs) (Fig. 4f).

### Humoral immune responses to chimeric RHDV-M2 VLPs

**Figure 5:**
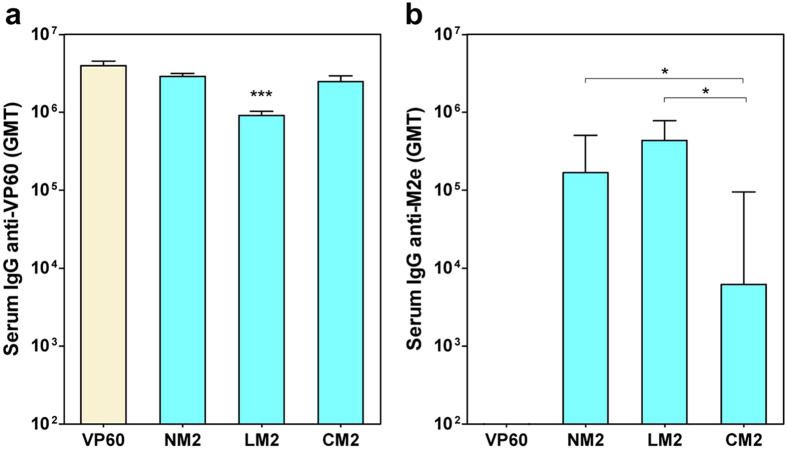
Humoral responses induced in mice by immunization with chimeric RHDV-M2 VLPs. Sera samples from groups of mice inoculated twice with the indicated VLPs were analyzed by ELISA. Serum IgG antibody titers were measured using RHDV VLPs to detect anti-VP60 antibodies **(a)**, or a synthetic peptide encompassing the M2e sequence **(b)**. The GMT was calculated for each group of mice. Error bars show the standard error of the mean. In **(a)**, statistically significant differences in anti-VP60 antibody titers, with respect to that corresponding to the VP60 group are shown as *P < 0.05, **P < 0.01, ***P < 0.001. In **(b)**, statistically significant differences in anti-M2e antibody titters between groups are shown as*.

Groups of five BALB/c mice were immunized twice intraperitoneally with 100 μg of each purified VLP emulsified in Montanide adjuvant. Blood samples were collected 14 days after boost and sera were tested for antibodies against VP60 protein (RHDV VLPs) (Fig. 5a) and M2e (a synthetic peptide) (Fig. 5b). Serum IgG antibody titers were measured by ELISA, and the GMT was calculated for each group of mice. All preimmune serum samples were negative (titer < 50) for IgGs against both antigens tested (data not shown). Control mice only receiving PBS with adjuvant also lacked specific serum IgG (titer < 50; data not shown). All mice immunized with RHDV or chimeric RHDV VLPs developed high titers of VP60-specific antibodies (GMTs ranging from 8.73 × 10^5^ to 3.83 × 10^6^). As previously shown with RHDV-FCV VLPs, the chimeric construct harbouring the foreign epitope at loop L1 of VP60 protein (LM2), elicited significantly lower VP60-specific antibody titers (*P* < 0.001) than native RHDV VLPs. All mice immunized with RHDV-M2 VLPs developed anti-M2e specific antibodies, whereas mice immunized with native RHDV VLPs (VP60) were negative (titer < 50). (Fig. 5b). Resembling the results described above for RHDV-FCV VLPs, the group of mice inoculated with chimeric construct LM2 (GMT = 4.36 × 10^5^) exhibited a higher GMT than that corresponding to group NM2 (GMT = 1.69 × 10^5^; although the difference was not statistically significant), and group CM2 (GMT = 6.15 × 10^3^; *P* < 0.05).

### Protection induced by RHDV-M2 VLPs against a lethal influenza virus challenge

**Figure 6:**
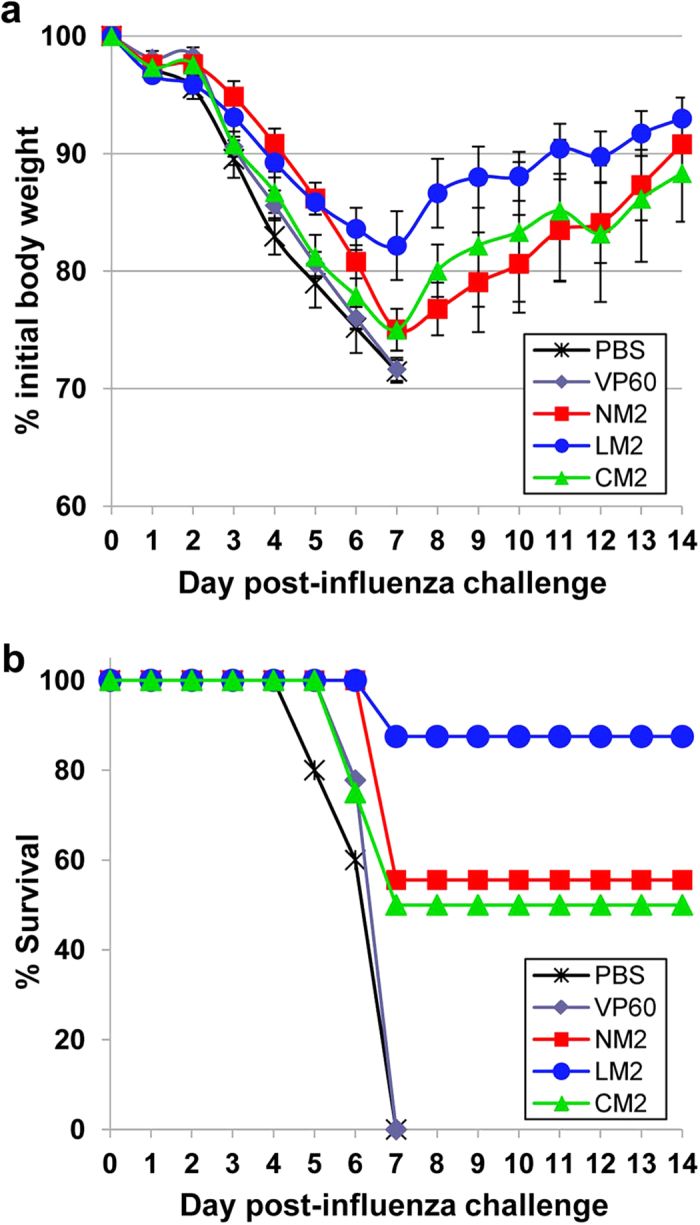
Protection against lethal challenge. Groups of mice immunized twice with the indicated VLPs (or PBS) were challenged intranasally with a lethal dose of influenza virus A/PR/8/34 (5 LD_50_). **(a)** Morbidity was assessed by daily monitoring body weight for two weeks. Values represent the mean weight of each group expressed as a percentage of the initial weight on the day of inoculation (100%). Error bars show the standard error of the mean. **(b)** Survival was monitored for 14 days and is expressed as the percentage of surviving mice.

Subsequently, we evaluated the protective efficacy of the RHDV-M2 VLPs in mice using a mouse-adapted influenza (PR8) challenge model. Groups of nine BALB/c mice were immunized twice by the subcutaneous route with 100 μg of each purified VLP emulsified in Montanide adjuvant. Mice were then challenged intranasally with a lethal dose (500 PFU/mouse) of influenza virus. One mouse from each group: LM2 and CM2, died before challenge. Body weight changes and survival rates were monitored for 14 days after virus challenge (Fig. 6). Mice belonging to the negative control group (PBS) and the group inoculated with native RHDV VLPs (VP60) showed a severe body weight loss from day 3 (Fig. 6a) and 100% succumbed to the influenza virus lethal challenge between days 5 and 7 (Fig. 6b). Mice immunized with NM2 and CM2 chimeric VLPs exhibited similar trends of morbidity and mortality. The mean body weight of both groups progressively decreased until day 7 post challenge, reaching average body weight losses of 24.97% (NM2) and 24.99% (CM2), and then gradually recovered to normal values. Between days 6 and 7 post challenge, 4 out of 9 mice from group NM2 and 4 out of 8 mice from group CM2, died (survival rates of 55.5% and 50%, respectively). In contrast, the group of mice immunized with LM2 chimeric VLPs exhibited a less drastic mean body weight loss (up to 17,84% by day 7), and mice rapidly recovered normal weight values, indicating mild disease. Only 1 out of 8 mice from this group died (survival rate 87,5%). Differences in body weight change and survival rates between the group of mice immunized with LM2 chimeric VLPs and the control groups (PBS and VP60) were statistically significant (*P* < 0.01). Taken together, LM2 chimeric VLPs successfully protected mice from a lethal challenge with influenza virus.

### Serum IgG titers against M2e induced by RHDV-M2 VLPs correlate with protection against influenza virus challenge

**Figure 7:**
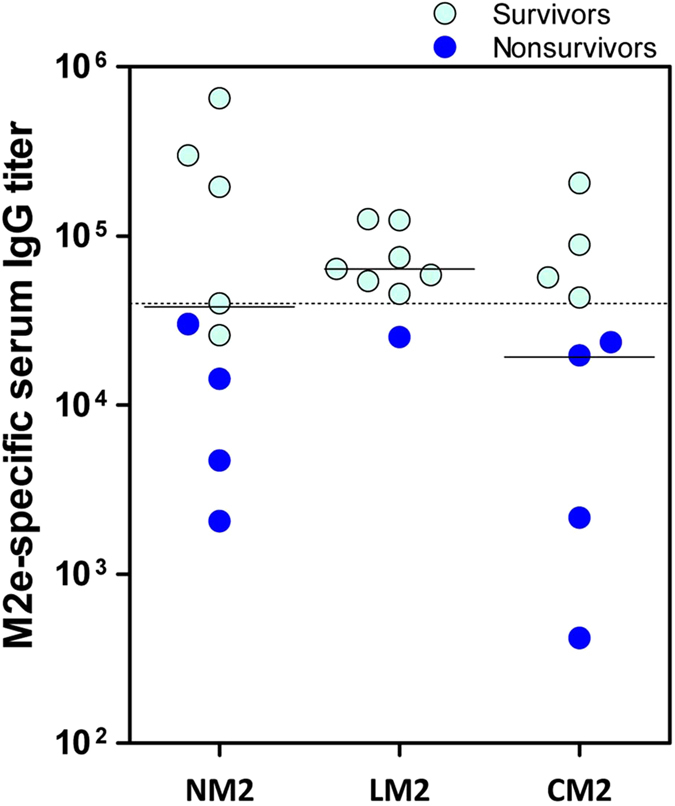
Serum IgG titers against M2e induced by RHDV-M2 VLPs correlate with protection against influenza A virus challenge. Sera samples obtained from the indicated groups of mice, just before the lethal challenge, were tested by ELISA for antibodies against M2e. Values represent the antibody titter of each individual mouse. The GMT was calculated for each group (solid lines). The dashed line depicts the antibody titer value of 39,000. All mice that elicited anti-M2e IgG titers above this threshold value survived the influenza virus lethal challenge.

Sera samples obtained just before challenge were tested by ELISA for antibodies against M2e (synthetic peptide) and GMTs were calculated for each group of mice (Fig. 7). Results obtained resembled those shown above (Fig. 5b), indicating that RHDV-M2 VLPs elicited similar immune responses in mice upon intraperitoneal (Fig. 5b), or subcutaneous (Fig. 7) administration. All mice immunized with RHDV-M2 VLPs developed anti-M2e specific antibodies (Fig. 7) whereas negative control mice (PBS) and mice immunized with native RHDV VLPs (VP60) were negative (titer < 50) (data not shown). Again, the highest GMT of IgGs against M2e corresponded to the group of mice inoculated with chimeric construct LM2 (GMT = 6.37 × 10^4^), which was higher than that corresponding to groups NM2 (GMT = 3.78 × 10^4^) and CM2 (GMT = 1.92 × 10^4^). Interestingly, the results demonstrated a clear correlation between the anti-M2e IgG titers elicited by chimeric RHDV-M2 VLPs in mice and the level of protection afforded against lethal influenza virus challenge (Fig. 7). Thus, all mice eliciting anti-M2e IgG titers ≥ 39,985, survived lethal challenge, while mice that developed anti-M2e IgG titers ≤ 30,161, succumbed to the influenza virus infection (except one mice from group NM2 which survived, having an anti-M2e IgG titer of 25,812). In light of these results, a set point of specific IgG titer against M2e of ≥39,985 could be considered a correlate of protection in this system. Additionally, this result provided an explanation for the marked differences observed in morbidity and survival rates, between the group of mice inoculated with LM2 and the groups of mice inoculated with NM2 and CM2. As shown in Fig. 7, immunization with LM2 promoted a more consistent and homogeneous humoral immune response, with 7 out of 8 mice (as well as the GMT of the group) exhibiting anti-M2e antibody titers above the observed threshold level associated with protection. In contrast, immunization with NM2 and CM2 promoted more heterogeneous responses, with around 50% of mice (as well as the GMTs of the groups) exhibiting anti-M2e antibody titers below the threshold level associated with protection.

## Discussion

Currently, there is a clear trend towards the establishment of VLPs as a powerful tool for vaccine development^32^,^33^. VLP-based vaccines have already been licensed for human diseases as well as for use in the veterinary field, and many more vaccine candidates are currently in late stages of evaluation^34^,^35^,^36^.

Our previous studies showed that RHDV VLPs are easily produced to high yields, are highly immunogenic and can be used as diagnostic reagents or vaccines for the control of RHDV in rabbits^25^,^28^,^37^,^38^. In addition, we have shown that RHDV VLPs are good platforms for inducing immune responses against inserted heterologous cytotoxic CD8+ T-cell epitopes and CD4+ T-helper epitopes, in mice and pigs^39^,^40^. Other studies have addressed the potential of RHDV VLPs as a platform for delivery of tumor-associated antigens, demonstrating their ability to elicit protective cellular immunity against tumors^41^. RHD VLPs are efficiently uptaken by dendritic cells^39^,^42^,^43^, which cross-present foreign T-cell epitopes via the MHC I pathway to initiate cytotoxic T-cell responses^40^,^41^,^43^.

In this study, we further extended the potential of RHDV VLPs as an excellent novel vaccine platform, demonstrating their ability to elicit potent protective humoral responses against inserted foreign B-cell epitopes. We have shown (i) that foreign B-cell epitopes can be incorporated at least at three different insertion sites, even simultaneously, without affecting the ability of RHDV VP60 to self assemble into VLPs, (ii) that RHDV VLPs tolerate the insertion of up to at least three tandem copies of a foreign epitope (62 amino acids) at a surface-exposed insertion site, two tandem copies (42 amino acids) at the N-terminal end of the capsid protein or one copy (22 amino acids) at the C-terminal end, (iii) that chimeric RHDV VLPs elicit potent protective humoral responses against inserted foreign B-cell epitopes, demonstrated by both, *in vitro* neutralization and *in vivo* protection against a lethal challenge.

The primary goal of this study was to develop chimeric RHDV VLPs eliciting humoral immune responses to foreign B-cell epitopes. A potential problem we faced was the reported high immunogenicity of native RHDV VLPs^24^,^37^, which might be detrimental, as the high level immune responses elicited by immunodominant regions of RHDV capsid could mask any specific responses directed to the incorporated foreign epitopes. With this in mind, in an attempt to increase the chances of success, we decided to evaluate different insertion sites, as well as preparing chimeric VLPs harbouring different copy numbers of the foreign B-cell epitope.

To perform this task two B-cell epitopes with different characteristics were selected. The first one was a newly described B-cell epitope derived from the capsid protein of FCV. The epitope is located at loop B’C’ within P2 subdomain of VP62 protein encompassing amino acid residues 440–459. It lies within one of the two hypervariable regions (HVR) identified in VP62 protein, the N-terminal HVR (aa: 426–460), which has been reported to be involved in FCV virus-receptor interaction^44^. It was identified by epitope mapping of neutralizing monoclonal antibodies obtained in our laboratory. Thus, it was anticipated that chimeric RHDV VLPs displaying this FCV epitope should be able to elicit a neutralizing immune response against FCV. Indeed, this was the case illustrating the applicability of RHDV VLPs as a platform for production of antibodies with desired specificity, directed against small peptides of choice. The second epitope used was M2e, a model antigen which has been successfully used by other VLP-based antigen delivery systems to protect mice against an influenza virus lethal challenge^30^,^45^. It was chosen because the mechanisms by which M2e-based immunogens provide protection against influenza virus infection have been analyzed in detail^46^,^47^,^48^. Antibodies raised against M2e do not induce neutralization, but rather act through antibody dependent cytotoxicity (ADCC) to limit and clear viral infection^47^.

Insertion site influenced the magnitude of humoral immune responses elicited against foreign epitopes displayed by chimeric RHDV VLPs. Incorporation at loop L1, within the P2 subdomain of VP60 protein, rendered the highest specific antibody titers (higher GMTs) against both B-cell epitopes assayed, and importantly, the most consistent humoral immune responses within the immunized groups (lower dispersion of the antibody titers induced), indicative of a more solid immune response. This fact seemed to be determinant in the different survival rate exhibited by mice inoculated with LM2, with respect to mice inoculated with NM2 or CM2 (see Fig. 7).

Several factors might account for the differences observed regarding the insertion site. Clearly, incorporation of foreign epitopes at loop L1 confers the highest surface accessibility to the inserted sequence, as compared to N or C-terminal ends of VP60 protein. Another relevant factor might be the immunodominant status of the region involved. Noteworthy, insertion site at loop L1 (between amino acid residues 306 and 307) lies within variation region V1 (amino acids 301–310), one of the seven highly variable regions defined on VP60 capsid protein^26^. In fact, V1 constitutes the most diverse region among RHDV isolates^49^. It is targeted by monoclonal antibodies which discriminate between classical RHDV and novel RHDV2 viruses^25^,^50^. Moreover, cell-and animal-based experiments with synthetic peptides encompassing loop L1 (amino acids 300–318) showed this region is involved in virus-host interactions and stimulates production of protective neutralizing antibodies^26^. Thus, previous reports indicated loop L1 region plays a critical role defining RHDV antigenicity and immunogenicity. Our results further reinforced the notion of an immunodominant status for loop L1, by means of incorporating foreign epitopes at this region. It not only resulted in high levels of antibodies against the inserted heterologous sequence (Figs 3b and 5b) but it also led to a significant reduction of antibody titers against VP60 protein (Figs 3a and 5a). In addition, the fact that antibody titers induced against FCV B-cell epitope by chimeric construct L1FCV were even higher than those induced by native FCV VLPs (i.e. epitope displayed in its natural context) (Fig. 3b), further highlighted the suitability of loop L1 region as insertion site for foreign B-cell epitopes. Finally, incorporation of foreign epitopes at loop L1 should lead to presentation of such epitopes as closed loops (i.e. with a constraint configuration), whereas incorporation of epitopes at the terminal ends of the capsid protein leaves the inserted peptide with either a free N or C-terminal end, thus allowing it to adopt a less constrained configuration and higher mobility. Previous studies focused on the relationship between the three-dimensional structure of an epitope inserted at the cowpea mosaic virus (CPMV) platform and its immunogenicity^51^, revealed that presentation as a closed loop is probably essential for good mimicry in the case of epitopes which adopt a constrained structure in their native context, while accurate structural mimicry is not an essential requirement in the case of antigens that act as linear epitopes and can be active even in a denatured form^52^,^53^.

Copy numbers of the foreign B-cell epitope per VLP monomer were shown to influence the immune responses elicited by chimeric RHDV VLPs. Increasing epitope copy numbers resulted in higher specific antibody titers (Fig. 3b) and a concomitant enhancement of the neutralizing activity elicited (Fig. 3c). The best results were obtained with constructs L3FCV and NLCFCV, both harbouring three copies of the foreign epitope per VP60 monomer, (540 copies per VLP). At least in some instances (N2FCV vs. N1FCV and L3FCV vs. L1FCV) the increased specific immunogenicity observed seemed to reflect a synergistic effect of tandem repeats over the immune response elicited. These results were in agreement with previous reports on the role of epitope repetitiveness and organization using different approaches: haptenated polymer molecules^54^, soluble carrier proteins^55^ or conjugated VLPs^56^, all of which highlighted the importance of high local epitope densities for induction of optimal B-cell responses.

In summary, using two different B-cell epitopes we have demonstrated that the RHDV-VLP display platform is able to induce a potent specific neutralizing antibody response or a protective immune response against a lethal virus challenge. Remarkably, RHDV VLPs were shown to be very tolerant not only in accepting foreign sequences at different insertion sites but also by incorporating them as several tandem repeats. The availability of at least three insertion sites per VLP monomer together with the tolerance to incorporate rather large heterologous sequences provides opportunities for versatile vaccine designs. The same antigen can be inserted at different sites and/or incorporated in tandem repeats, as was done in this study, to increase the desired specific immune response. Alternatively, different antigens could be incorporated to the RHDV VLPs, resulting in a multivalent vaccine. Interestingly, since our previous work had shown that in order to induce cellular immune responses against foreign T-cell epitopes, incorporation at the N-terminal end of VP60 protein was the best choice^39^, our overall results open the possibility of incorporating simultaneously foreign B and T cell epitopes, at loop L1 and the N-terminus of VP60 protein, respectively. The combination of different types of epitopes (B, CD4+, and CD8+) incorporated at different sites of RHDV VLPs, paves the way for developing highly efficient multimeric vaccines, able to stimulate the different branches of the immune system.

It has been shown that pre-existing antibody responses against VLPs may exert detrimental effects on the efficacy of chimeric VLP-based vaccines^57^. This is a potential problem faced by VLP vaccine platforms derived from viruses which are highly prevalent among humans or relevant livestock species^57^,^58^. Since RHDV has a narrow host-range restricted to rabbits^23^, no pre-existing immunity against RHDV capsid is expected in other species, thus avoiding the problem for the first use of the RHDV VLP-based platform. However, pre-existing immunity might adversely affect further uses of this VLP platform for different indications. Additional studies are required to clarify this issue. At this respect, it is assumed that no single VLP platform will be able to meet all vaccination needs^59^. Therefore, the continued parallel development of multiple VLP platforms is expected to collectively allow to cover the requirements of individual vaccines. The RHDV VLP-based platform reported here represents a suitable choice of delivery system, which further extends the arsenal of VLPs available for new vaccine development.

In conclusion, our results demonstrate RHDV VLPs constitute an attractive platform for wide application in new vaccine development, both in human and the veterinary field.

## Methods

### Virus, cells and mice

Derivatives of *Autographa californica* nuclear polyhedrosis virus (AcMNPV) were used to obtain the recombinant baculoviruses expressing RHDV VLPs. Baculoviruses were propagated in *Trichoplusia ni* High five cells (H5) grown in monolayer cultures at 28 °C in TNM-FH medium (Sigma-Aldrich), supplemented with 5% fetal calf serum (Gibco)^60^. Feline calicivirus (FCV), Urbana strain, kindly provided by K.Y. Green (NIAID, NIH, Bethesda, MD, USA), was used to generate FCV-VLPs and to perform serum neutralization tests. Influenza A virus mouse-adapted PR8 strain (A/PuertoRico8/34, H1N1), kindly provided by A. Nieto (CNB-CSIC, Madrid, Spain) was used in the murine challenge experiments.

The immune responses induced by the different chimeric VLP constructs were assessed in mice (6- to 7-week-old female) (Harlan). C57BL/6 mice were used in experiments involving RHDV-FCV VLPs and BALB/c mice were used in experiments with RHDV-M2 VLPs.

### Generation of recombinant baculovirus transfer vectors

Plasmid pHAPhSubGB, containing the coding sequences of proteins VP60 and VP2, and the 3′ untranslated region of RHDV (strain AST/89, GenBank accession code Z49271)^39^ was used as starting point. To generate plasmid vectors corresponding to VP60 constructs incorporating foreign amino acid sequences we first engineered unique BamHI restriction sites by site-directed mutagenesis at: amino acid positions 2 and 3 (plasmid pHAPh2GS); positions 306 and 307 (plasmid pHAPh306GS); and immediately before the VP60 stop codon (pHAPh580GS). The next step was the insertion of the foreign sequences of interest. For this, DNA fragments were generated by annealing the corresponding synthetic phosphorylated oligonucleotides leaving BamHI compatible ends: NeuFCV22F/NeuFCV22R, encoding the 22 amino acid sequence GSGNDITTANQYDAADIIRNGS, encompassing the FCV neutralizing B-cell epitope used in this study, and oligonucleotides sM2F/sM2R, encoding the 22 amino acid sequence GSLLTEVETPIRNEWGCRCNGS, encompassing M2e. The annealed primers were subsequently ligated into the corresponding plasmids (pHAPh2GS, pHAPh306GS and pHAPh580GS) previously linearized by BamHI digestion and dephosphorilated. After ligation and transformation, bacterial colonies were analyzed by PCR to identify plasmids containing one, two or three tandem inserts in the correct orientation. All the inserted sequences in the resulting recombinant plasmids were verified by sequence analyses.

### Generation of recombinant baculoviruses

All recombinant baculoviruses were generated using the flashBACULTRA expression system (Oxford Expression Technologies) following the manufacturer’s instructions.

### Expression and purification of recombinant RHDV VLPs

H5 cell monolayers were infected with recombinant baculoviruses at a multiplicity of infection (MOI) of 10. After incubation (4 days, 28 °C), infected cells were dislodged into the growth medium and collected. Suspensions were then washed three times with 0.2 M phosphate-buffered saline for VLPs (PBS-V, consisting of 0.2 M sodium phosphate, 0.1 M NaCl, pH 6.0). Next, pellets were resuspended in distilled water, subjected to mild sonication and treated with DNAse I (Roche Applied Science) for 1 h at room temperature. Subsequently samples were adjusted to 2% Sarkosyl (sodium N-lauroylsarcosine, Sigma), 5 mM EDTA in PBS-V, and incubated overnight at 4 °C. Cell lysates were then clarified by low-speed centrifugation and the supernatant was centrifuged at 27,000 rpm for 2 h using a Beckman SW28 rotor. The pelleted material was resuspended in PBS-V, extracted 2–3 times with Vertrel XF (Fluka, Sigma-Aldrich), and centrifuged at 27,000 rpm for 2 h using a Beckman SW28 rotor. The pellets were finally resuspended in PBS-V containing protease inhibitors (Complete, Roche) and stored at 4 °C. Protein concentrations of the VLP preparations were determined with BCA protein assay kit, Pierce, Thermo Scientific.

### SDS-PAGE and Western blot analyses

Samples were boiled for 5 min in protein dissociation buffer containing 5% (vol/vol) β-mercaptoethanol, 2% (wt/vol) sodium dodecyl sulfate (SDS), 10% (vol/vol) glycerol, 80 mM Tris-HCl (pH 6.8), and 0.01% (wt/vol) bromophenol blue. Proteins were resolved by SDS-polyacrylamide gel electrophoresis (PAGE) and were visualized by Coomassie brilliant blue staining.

For Western blot analyses, proteins were transferred from gels onto polyvinylidene difluoride (PVDF) membranes by standard blotting procedures. Membranes were saturated (overnight, 4 °C) with PBS–5% (wt/vol) nonfat dry milk and incubated (1 h, 37 °C) with either a rabbit hyperimmune serum against RHDV to detect VP60 protein, or monoclonal antibodies directed against the inserted foreign epitopes, as indicated. After several washes with PBS–0.05% Tween 20 (PBS-T), membranes were incubated (1 h, 37 °C) with HRP-conjugated goat anti-rabbit IgG (Pierce) or HRP-conjugated goat anti-mouse IgG (Invitrogen). Membranes were then washed extensively with PBS-T and developed with peroxidase substrate (0.05% 4-chloro-1-naphthol, 0.08% H_2_O_2_ [30%], and 20% methanol in PBS).

### Transmission electron microscopy

Samples (approximately 5 μl) were applied to glow discharged carbon-coated grids for 2 min and negatively stained with 2% (wt/vol) aqueous uranyl acetate. Micrographs were recorded with a Jeol 1200 EXII electron microscope operating at 100 kV at a nominal magnification of x40,000.

### Mice immunization

Groups of five mice were inoculated by the intraperitoneal route at days 0 and 21, with 100 μg of the indicated VLP constructs emulsified in Montanide ISA 50V2 (Seppic, France), and sacrificed at day 35. A negative control group was inoculated with PBS plus adjuvant. Blood samples were collected at day 0 (before priming), day 21 (before boost) and at day 35.

### Detection of specific anti-RHDV-VLP antibodies by ELISA

Polysorp 96-well ELISA plates (Nunc) were coated with purified RHDV VLPs (300 ng/well) diluted in 0.05 M carbonate-bicarbonate buffer (pH 9.6), and incubated overnight at 4 °C. Plates were blocked with PBS–5% skim milk (5% BLOTTO) for 2 h at 37 °C, washed six times with PBS-T and incubated with serial three-fold dilutions of each serum sample prepared in 5% BLOTTO, for 2 h at 37 °C. Two control samples (serial dilutions) were added to each plate: a monoclonal antibody specific against RHDV VP60 protein^25^, and a serum sample known to lack VP60-specific antibodies. The test sera samples were analyzed in parallel in wells lacking antigen to determine background binding. After six washes with PBS-T, the plates were incubated at 37 °C for 45 min with HRP-conjugated goat anti-mouse IgG (Invitrogen) at a 1/3,000 dilution in 5% BLOTTO. Finally, plates were extensively washed with PBS-T and colour reaction was developed with peroxidase substrate (4 mg/ml *o*-phenylenediamine and 0.08% H_2_O_2_ [30%] in 0.05 M phosphate-citrate buffer [pH 5.0]). Reaction was stopped by addition of 3N H_2_SO_4_, and colour development was recorded at 492 nm. End point titers were reported as the reciprocal of the highest dilution that had an absorbance value greater than or equal to 0.2 O.D units above background (absorbance of wells lacking antigen, which were consistently in the range of 0.035–0.055, and never exceeded 0.085).

### Detection of specific anti-FCV capsid and anti-influenza M2e antibodies by ELISA

Sera samples were examined by ELISA assays using synthetic peptides as antigens: peptide GSGNDITTANQYDAADIIRN, encompassing the sequence of the FCV B-cell epitope (Pompeu Fabra University, Barcelona, Spain) and peptide MSLLTEVETPIRNEWGCRCNGSSD comprising the M2e 24 amino acid sequence (New England Peptide). Assays were performed in 96-well high binding plates (Corning), coated with 1 μg/well of synthetic peptide diluted in PBS buffer overnight at 4 °C. The protocol was completed as described above for RHDV-specific antibody ELISAs, except that reactions were developed with 4% 3, 3′, 5, 5′ tetramethylbenzidine (TMB) peroxidase liquid substrate containing 0.02% H_2_O_2_ [30%]. Reactions were stopped by addition of 1,8 N H_2_SO_4_ and colour development was recorded at 450 nm.

### FCV seroneutralization assay

Serum samples diluted 1/20 in EMEM were incubated with 150–200 PFU of FCV (Urbana strain), for 1 h at 37 °C in a final volume of 200 μl (M96 plates). Similar samples were prepared in the absence of serum as controls. Subsequently, the samples (triplicates of each serum) were added to monolayers of feline CRFK cells and incubated for 1 h 30 min at 37 °C in a total volume of 0.8 ml (M6 plates). The inocula were removed and plaque assays were performed under an overlay of 1.0% low-melting-point agarose in complete EMEM^61^, until visible plaques appeared (1–2 days) upon staining with crystal violet. Percent of plaque reduction was calculated by comparing the number of viral plaques present in each sample relative to the amount of viral plaques formed in the absence of serum (means of triplicates).

### Influenza virus challenge experiments

Groups of 9 mice were inoculated by the subcutaneous route at days 0 and 21, with 100 μg of the indicated VLP construct emulsified in Montanide ISA 50V2 (Seppic, France). An additional group was inoculated with PBS plus adjuvant. Blood samples were collected at days 0 (before priming), 21 (before boost) and 35 (before challenge). At day 35, mice were challenged intranasally with a 5 LD_50_ dose (500 PFU/mouse) of mouse-adapted influenza virus PR8. Morbidity was assessed by daily monitoring body weight for two weeks. At day 49, surviving mice were humanly sacrificed.

### Data and statistical analysis

Values were reported as geometric mean titers (GMT) ± standard error of the mean. Statistical analyses were performed using GraphPad Prism 5.00 for Windows (GraphPad Software, San Diego, CA, USA). Antibody titers were compared among groups of mice using one-way analysis of variance (ANOVA), followed by Tukey’s or Dunnett’s post-hoc comparison tests. Statistical significance for Kaplan-Meier survival curves was calculated with a log-rank (Mantel-Cox) test. All *p* values are two sided and p ≤ 0.05 were considered significant. In figures, *p* value criteria are assigned as *p < 0.05, **p < 0.01, ***p < 0.001.

### Ethics statement

Mice were maintained at the CISA-INIA animal facilities according to European Union and national guidelines for animal experimentation. The study received prior approval from the Ethical Committee for Animal Experimentation (CEEA 2013/009) and Biosecurity Committee of INIA (CBS 2013/006). All experimental procedures were conducted in accordance with protocols approved by animal welfare authorities, with the reference PROEX 47/13.

## Additional Information

**How to cite this article**: Moreno, N. *et al*. Rabbit hemorrhagic disease virus capsid, a versatile platform for foreign B-cell epitope display inducing protective humoral immune responses. *Sci. Rep.* **6**, 31844; doi: 10.1038/srep31844 (2016).
